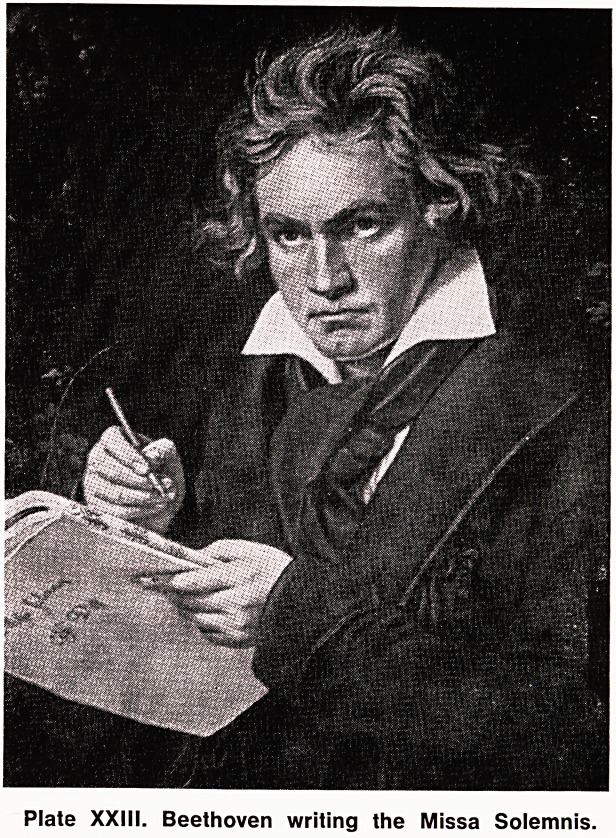# Beethoven (Muss es sein? Es muss sein!)

**Published:** 1971-07

**Authors:** Beryl D. Corner

**Affiliations:** Presidential Address to Bristol Medico Chirurgical Society October 1970


### Beethoven

MUSS ES SEIN? ES MUSS SEIN!

(The difficult Question—Must it be? It must be!)

by

**Beryl D. Corner, M.D., F.R.C.P.**

Presidential Address to Bristol Medico Chirurgical Society

October 1970

"The child is father of the man" is the philosophy of the paediatrician, hence the recent trend of intense interest in child development, which embraces the study of factors which influence the growth of the body, mind, personality and spirit from conception to maturity and ultimate achievement. As practitioners of medicine it is natural that much of our effort should be used in the diagnosis and care of those who have little potential or fail to reach their optimum in few or many respects. By contrast, how rarely do we interest ourselves in the development of the highly gifted, and the genetic and environmental influences that lead the rare genius to the achievement of excellence!

The qualities of genius have been variously defined: "Originality and uniqueness of vision so far above the ordinary as not to be explained in the normal terms of genetic inheritance; character and singleness of purpose are as important as the more sublime and visionary elements" (Tennyson, 1970). "An extra quality which liberates man from external circumstances". (Burnett James, 1960).

This year, the world is celebrating the bicentenary of the birth of Beethoven, the greatest musical genius of all time in the opinion of many.

His influence is as strong as ever and still musicologists are devoting their lives to the study of his work. His genius was recognised during his life, and his development and achievements are of considerable psychological and medical interest. Legend and interpretation fill the enormous bibliography about him. This glimpse of the development and achievements of Beethoven as seen by a paediatrician is based on a study of the original writings of his contemporaries, Wegeler and Ries and others, collected letters (Emily Anderson), sketch books and conversation books, Thayer's and Schiedermair's biographies. Beethoven presents the eternal dilemma of the extent to which the achievements of creative genius are inevitable and how much do they owe to environmental influences.

**Beethoven's birth**

Beethoven was born in Bonn, in a little attic of a garden annexe to No. 515 Bonngasse. The exact date is unknown but as he was baptised, Ludwig, on 17th December 1770, either the 15th or 16th December are likely to be his birthday, since it was usual for Roman Catholic baptism to be carried out within 24 hours of birth, owing to the high neonatal mortality rate. The godparents were his grandfather Ludwig van Beethoven, and the next door neighbour, the wife of the Clerk to the Elector's wine cellar. The christening feast was held in her house because the young Beethoven's flat was too small. At the time of his birth his father was 30 and his mother aged 24.

**Genetic Influences**

Families named Beethoven can be traced to towns and villages round Antwerp in central Flanders in the 15th and 16th centuries, working as farmers, craftsmen and occasionally shopkeepers, who tended to settle in the prosperous towns of Mechelin and Leuwen. The first musical member of the family was Ludwig van Beethoven born in 1712 to Michael, a carpenter living in Mechelin, whose wife came from Rhineland, but of their four children only two survived. Ludwig the younger son, showed signs of musical talent and was sent for training to the Cathedral Choir School in Mechelin; he also played the organ. At 19 years, he went as bass singer to Liège Cathedral, and after a year was invited to join the choir of the Elector of Cologne in Bonn where his brother Cornelius had already gone to trade as a chandler. A different racial genetic influence was introduced when Ludwig married Marie Josefa Pohl, a Rhenish girl whose ancestors probably came from Bavaria and Eastern Germany, a part of Europe with a reputation for musical talent.

A successful musician, Ludwig had high professional and social prestige in Bonn, and in 1761 became Kapellmeister to the Elector Maximilian Friedrich and responsible for all music at the Court, which he served for 41 years. Their two first children died very shortly after birth, and only Johann born in 1739 who became the father of the composer, survived. Their home was an apartment in the Fischerhaus, owned by a family of master bakers. As a side-line Ludwig carried on a wine business and this may well have been the undoing of his family. In later life his wife became a chronic alcoholic, and her last years were spent in an institution where she died in 1775. As a result of family worries "This highly respected man, so good-hearted in his social relationships became embittered in his old age".

Ludwig van Beethoven's only surviving son, Johann, at a very early age showed musical talent. Following the pattern of his father's early life he was trained as a chorister and also learnt to play the klavier and violin. It was customary for Court musicians to be appointed initially in an honorary capacity as an "Accessit", and at the age of 16 years Johann was thus appointed as a tenor at the Elector's Court. He earned his living as a popular and successful teacher of klavier and singing to many eminent families including those of foreign diplomats and courtiers; perhaps because of the small financial reward he received many gifts of barrels of Rhenish wine. His first very small Court salary (100 Thalers) was not awarded until the age of 24. A restless man, inclined to be unstable and moody, but in his work undoubtedly scrupulous and meticulous, he lacked his father's quality both as a person and a musician, so the family remained very poor.

Johann's marriage to Maria Magdalena Leym, took place in 1767 quietly at St. Remigius Church, Bonn. She was a young widow in her 20th year from Ehrenbreitstein. Her father was Chamberlain to the Elector of Trier and well connected with people prominent in business and political circles. Her first husband died when she was 18 and their only son at 5 months. The couple acquired the small garden flat at the back of the house of Clasen the lacemaker, and father Ludwig moved to lodgings near them in Bonngasse, which was the favourite residential area for musicians, artists, and the intelligentsia. Simrock, horn player in the Elector's orchestra opened a music publishing house there which was most opportune for the publication of the young composer's early works. Ludwig Maria, their first son born in April 1769, died after a few days.

**The ecology of Beethoven's childhood**

"A child's environment comprises all the external conditions and influences under which he lives" (Apley, 1964). The environment of Beethoven's childhood provided him with almost the whole spectrum of emotional experience which was vital for the development of his creative genius, including much family illness, bereavement and turbulence, but affection and some success too. At an early age he showed that singleness of purpose which breaks through all difficulties of environment.

He was born in the right place at the right time, at Bonn in the second half of the 18th century. This was a city of 8,000 inhabitants situated in a lovely part of the Rhineland, facing the seven hills with their forests and fertile valleys. The Electors of Cologne, Princes of Church and State had made it their residence since the thirteenth century. Bonn was surrounded with conflicts and wars, but as Archbishops, the Electors kept no armies so their interests and wealth were expended on cultural pursuits. In the first half of the 18th century Elector Clement August encouraged the musical activities so that by the time of his death in 1761, Bonn had a high reputation for music and the arts, and philosophy. He was succeeded by Elector Maximilian Friedrich who was even more ambitious that Bonn should rival Vienna as a cultural centre.

The young child is immediately encircled by his home and family. What a precious and longed for child Ludwig must have been for these parents! During the most sensitive learning period of the first three years he was fortunate in being an only child. From the time of his conception they must have determined to try to make him a musician. In their attic flat pupils and musicians came to play and study music with his father. His grandfather lived so near that he was often in the home, and despite the fact that he was rather an embittered old man he helped to create an atmosphere of music in the Beethoven home until he died suddenly from a stroke in 1773. Ludwig understandably regarded him with great affection and his portrait by Radoux hung in the composer's room all his life.

Beehoven referred to his father as "My good father". Johann was a young striving musician, a well liked and efficient teacher who found it difficult to provide for his increasing family. In his early years he was concerned for the welfare of his children but later he became steadily more moody and irresponsible and with bad companions went on drinking bouts and gradually lost interest in his family. His wife, Magdalena, was a tall slender woman with a equeline nose, honest eyes, and a sad face, rarely displaying any vivacity. Although saddened by a series of bereavements in her own family she obviously had a great capacity for forming a warm, happy relationship with her children. When she died Beethoven referred to her as "such a good kind mother to me and indeed, my best friend. Oh! who was happier than I, when I could still utter the sweet name of mother, and it was heard and answered". (Anderson). The composer probably owed a great deal to the sweet piety and intelligence of his mother and she was the important influence of his early years.

After his father's death, Johann's finances were precarious. He applied for an increase in salary to the Elector, but this was refused although the pension for his mother continued. During the next three years two more children were born, Casper Anton Carl in 1774 and Nicholas Johann in 1776. In all they had 7 children of whom only Ludwig, Carl and Nicholas Johann survived infancy. Twice more the family moved house before Ludwig was six, and finally made the Fischerhaus their permanent home. This was fortunate as the Fischer family became close friends and in the Fischer Manuscript recorded many incidents of Ludwig's childhood. Poverty surrounded Ludwig throughout his childhood and much of his adult life, he always found it difficult to manage finance or his household affairs, particularly as he grew older.

**General education**

His education was on the usual lines of those days. Like his father he attended the public Roman Catholic elementary school which taught Latin and a little French. His early education was sketchy, he always wrote badly, even simple arithmetic was too difficult and he never mastered multiplication. However, he developed a real love of poetry and reading. He left school at 11 years to earn his living just as his father had done, and music took precedence over everything else.

As he grew older he realised the necessity for education to broaden his experience. Many opportunities were available to him in Bonn to meet poets, philosophers and those associated with the intelligentsia and with the Court and University. "I have not the slightest pretension to what is properly called erudition yet from childhood I have striven to understand what the better and wiser people of every age were driving at in their works" (Anderson). He participated in student discussions and as time went on he made many influential friends.

In appearance he was a short stocky boy with dark hair and grey-blue eyes. Dr. Mueller described him: (Thayer) "a shy and taciturn boy the necessary consequence of the life apart which he led, observing and pondering more than he spoke, and disposed to abandon himself entirely to the feelings awakened by music and (later) by poetry and to the pictures painted by fancy"! He often lay on his attic bed looking out on to the river and the lovely seven hills beyond. Since early childhood he had loved to be alone in the countryside, so his leisure was spent walking there, studying natural events and going for trips on the river with his father. He occasionally played with his brothers and neighbour's children, and stories suggest that he enjoyed the usual boyish pranks.

**Musical Education**

This started in his 4th year as soon as he was able to reach the keyboard of the klavier, which necessitated standing on a wooden bench. His father began to teach him and soon recognised his talent, using stern methods and conventional technique for the harpsichord as expounded by the Italian and German keyboard teachers early in the century, which gave little scope for light and shade of expression. The child was often in tears but made to continue his studies unremittingly. He also learnt to play the violin for which he had little aptitude, often playing out of tune with wrong notes so that "even the flies and spiders fled from his bad scraping": (Schindler, 1840).

**Plate XX. Beethoven's clavichord, on which he learnt to play.**

His father being so poor sought to exploit the child as an infant prodigy following the example of Mozart 10 years previously. When he was in his 8th year, his father arranged for him to play at a concert in Cologne. As the boy looked small he falsified his age to 6 years. The programme of this concert is not recorded, but it is very likely that it included klavier works by C. P. E. Bach (example, Solfeggietto) and possibly the early Italian composers, Scarlatti and Corelli. Ludwig was no infant prodigy as he lacked Mozart's great facility for quickly absorbing different types of music.

Court musicians who lodged with the family played a considerable part in his musical education. Among these was Pfeiffer, an oboe player who gave him lessons on the klavier and also taught him to accompany the oboe; unfortunately Pfeiffer became his father's drinking companion and encouraged him to go on drinking bouts at night. The violinist Rovantini, a cousin of his mother, gave him his first lessons on the violin. In his 9th year he probably took organ lessons with van Eeden, the elderly Court Organist, and certainly from the Franciscan Friar, Willibald Koch, who allowed him to play the organ for Mass at the nearby Minorites Monastery.

Christian Gottlob Neefe came to Bonn in November 1779 to become Court Organist. Financial stringency in the Beethoven household made it essential that Ludwig should quickly become a wage earner; another child had been born in 1779 who died a few days later, and a fifth child in 1781. Johann thought his son could earn more quickly as an organist and asked Neefe to teach him. This was the first opportunity for Ludwig to receive training from a really competent teacher. He not only drilled him in "the old testament for the keyboard", the 48 Preludes and Fugues of J. S. Bach (example Fugue in C Major, No. 1) but, recognising the potential of his pupil as a composer, taught him thorough-bass and counterpoint, an essential basis for musical composition.

From his earliest childhood, Beethoven showed a defiance of convention and an urge to make his own music. He was constantly chastised by his father as he rebelled against practising music which did not give him emotional satisfaction. He was frustrated by the lack of singing tone in the klavier and the music of J. S. Bach and C. P. E. Bach was too precisely constructed and required too much discipline in execution to allow the boy to express himself adequately. This may account for the rare use of the fugue form in Beethoven's compositions until his later years.

The organ provided more opportunity for improvisation and when he was in his 10th year the story is told that he amazed the church choir and orchestra by his remarkable improvisation at Mass on a theme in the prelude to the Creed, which continued for much longer than was customary. This seems to be the first public glimpse of his potential genius as a composer.

Neefe was not only a fine musician but well educated as a philosopher and a very good friend to his pupil. Recognising the boy's genius, he encouraged his efforts at composition at age 11 years by arranging publication of "The theme and nine variations on a March by Dressler". This work was particularly interesting as a foretaste of thing to come. The form, that of theme and variations is one which the composer used so much in many differing types of composition that it almost became his own. The key, C minor, seems unusual for a little boy but is used in some of his great compositions later, e.g. the 5th Symphony and the Pathétique Sonata Opus 13. The march rhythm was a forerunner of the famous funeral marches of the pianoforte Sonata Opus 26, and the Eroica Symphony.

Neefe was a philosopher who related music to moral and spiritual life. He awakened his pupil's critical sense and aroused his interest in reading philosophy. This almost constant contact was an important influence, giving the boy that urge for virtue and goodness which was to become a lifelong obsession and which he only fully expressed in his later works. At the age of 11, Beethoven deputised for Neefe, while he was away on a tour, as cembalist to the theatre orchestra which meant that he acted as conductor and accompanist on the harsichord or klavier. This provided opportunity not only to learn to sight-read difficult scores but to become acquainted with popular Italian,

French and German operas. At thirteen, he was officially appointed assistant court organist to Neefe. Neefe's testimonial to the Elector reads: "Louis van Beethoven, son of the tenor singer, a boy of 11 years and of most promising talent. He plays the klavier very skilfully with power, reads at sight very well, and to put it in a nut shell plays chiefly the well-tempered clavichord of J. S. Bach. This youthful genius is deserving of help to enable him to travel. He would surely become a second W. A. Mozart were he to continue as he has begun". (Thayer, quoted from Cramer).

His skill at composition was developing and his works were often too difficult for him to play. In a childlike way he said "I will be able to play them when I am bigger". At this age his second published work, three sonatas for klavier, was dedicated to Maximilian Friedrich. Whilst being strongly reminiscent of the klavier works of Mozart and Haydn, Beethoven's own characteristics began to show, especially in the use of dramatic chords and dynamics, *fp* and sudden sforzandos. Beethoven's admiration for his teacher was expressed in a letter to him: "I thank you for the counsel which you gave me so often in my progress in my divine art. If I ever become a great man, you shall have a share of the credit" (Thayer).

**Adolescence**

During his early teenage period Beethoven showed a developing capacity to make close friendships and for the first time with boys of his own age. To help family finances he started to give klavier lessons. This took him to the household of Hélène von Breuning and her family. Her husband was a Court Councillor who had perished in the great palace fire in 1777 so she lived with her sister and brother-in-law who were important dignitaries of Court and Church in Bonn.

Beethoven owed a great deal to the friendship of the Von Breuning family. In their cultured home he learnt about social behaviour. Frau von Breuning treated him as her son and often attended to his clothing, appearance and manners. They had many visitors among whom was Franz Wegeler, a medical student who eventually married Eleanore, the eldest daughter. Dr. Wegeler became his lifelong friend and one of his most authentic biographers. Count Waldstein, another regular visitor greatly admired Beethoven and later gave him financial support to visit Vienna.

In 1784 disaster overtook Bonn, The Elector died and was succeeded by Elector Maximilian Franz, the last holder of this title. The Rhine flooded extensively, compelling the Beethovens to move their home and little brother, Franz George died at 2½ years. Elector Maximilian Franz brought a "new look" to Bonn. The Church and music were less important than academic development and big social changes were pending with the growth of the Liberation movement in France and Rhineland. In 1786 he gave his residence to the University of Bonn, the faculties included Theology, Philosophy, Medicine and Law. Beethoven later entered the new University to study philosophy, matriculating in 1789, and developed a lively interest in student activities, especially the new philosophy of the equality and brotherhood of man. He loved poetry and literature, and was much influenced by the writings of Kant and Schiller, as well as the classics.

In the meantime the fortunes of the Beethoven family were deteriorating. Magdalena had another child in 1786 after which she became ill with tuberculosis. Johann's depression increased, he became irresponsible and consoled himself with alcohol continuously. The story is told that the "Fate knocking at the door" theme derived from the many occasions when Ludwig and his friend Stephan von Breuning went round the streets at night knocking at doors to find him and take him home in a drunken state.

**Plate XXI. Beethoven, age sixteen years.**

1787 was a turning point in the adolescent Ludwig's professional life. He had for some time wanted to have lessons with Mozart, so it was arranged that he should travel to Vienna to meet Mozart. On April 7th he reached Vienna. Mozart was not at first impressed with his playing but when Beethoven asked him to give him a theme for improvisation, he did so well that Mozart commented "keep your eye on him, some day he will give the world something to talk about." Beethoven may have had a few lessons with Mozart but after two weeks he was summoned urgently back to Bonn, because of anxiety concerning his mother's health. The weather was bad, he was short of money and had a difficult journey which resulted in his first serious illness. There is a good deal of evidence that all through childhood he had a tendency to recurrent abdominal pain and diarrhoea, probably of psychosomatic origin; although some attacks might have been due to infective gastro-enteritis. He caught a severe respiratory infection on this journey and became depressed. He wrote "My longing once more to see my dying mother overcame every obstacle and assisted me in surmounting the greatest difficulties. I found my mother still alive but in the most deplorable state, and after much pain and suffering in July 1787 she died." His first attack of asthma followed and lasted for some weeks. A fear of consumption added to his melancholy.

The Elector's economies began to cause drastic changes in the Court musical arrangements. Beethoven's father now aged 44, with three sons and an infant daughter, is described as "having a very stale voice, has long been in the service, is very poor and of fair deportment'. Neefe was recommended for dismissal on the grounds that he was a Protestant, a foreigner and not particularly well versed as an organist; whilst Ludwig found favour "as being of good capability, still young, of good and quiet deportment and poor". After some persuasion the Elector reduced Neefe's salary but did not dismiss him and Ludwig was promoted to his post.

Beethoven's 17th year ended in great sadness, "Fate is not propitious to me here in Bonn". His sister Margaretha died aged 1 year. His two brothers were in early adolesence, Carl training for music and Johann as an apothecary. As his father by now was a hopeless wreck, Beethoven took over full responsibility as the head of the family. He applied to the Elector for his father's salary and shared this with him. He also became a viola player with the Court Orchestra for four years which gave him invaluable experience of orchestral repertoire and performance, and in 1789 he was appointed Court Pianist. The inadequacy of his training caused him increasing frustration as he was aware that Haydn and Mozart had both composed with remarkable facility whereas he laboured slowly with tremendous effort and many corrections before he was satisfied with his work. This Court appointment gave him a breathing space so that he was able to broaden his musical experience and was a blessing in disguise.

His songs and compositions for small groups of instrumental players and pianoforte were popular as were his exciting improvisations, and his performance on the klavier or pianoforte began to gain recognition. The habit of writing down his ideas whenever they came in sketch books which were subsequently to become so famous was now developing, and at times his fervour for musical composition seemed to possess him so that he withdrew completely into a state which Frau von Breuning called his "raptus". He was however so self-critical of his work that none of the writings of the Bonn period were given opus numbers. Musical genius depends on the use of intellect to express emotion, experience and ideas. During these 21 years he had accumulated a large store of very diverse experience and acquired an outstanding capacity for work but his intellectual power was still to be manifested.

The development of the pianoforte was another fortunate accident of time for Beethoven. To express his ideas he needed an instrument on which he could control the dynamics with his fingers, an impossibility with plucked strings only. The pianoforte and fortepiano enabled him to develop a new style of legato and cantabile playing and an entirely different technique for the keyboard from any of his predecessors. Sudden, as well as carefully graduated changes of tone volume control enabled the master to produce the beautiful and dramatic contrasts which characterised his work.

In 1790 Haydn came to Bonn and after hearing Beethoven play the pianoforte persuaded him to go to Vienna to continue his studies. Mozart died in 1791, so with the great help of his influential friend Count Waldstein he was given leave from the Court in 1792 with his salary continued "To receive Mozart's spirit at the hands of Haydn". His father died in 1792 and Beethoven left for Vienna never to return to his native city.

**Vienna**

In Vienna, a new life started for the 21-year-old boy, For three years he studied pianoforte and violin playing and composition, but was disappointed with Haydn's teaching. An immature, moody, socially eccentric, rough and ill-mannered young man although obsessional about personal cleanliness, his way of life was disorderly but in his musicanship he was self-confident, and outspoken in his political views. In 1795 he made his real debut as master of the pianoforte. Opus numbers were then given to his works which included the piano trios, some vocal works and chamber music.

Bonn meanwhile had undergone a revolution as Napoleon's army had overrun the city, and the Elector was deposed. Beethoven's salary ceased, so at the early age of 25 he was suddenly forced to become a free-lance composer and pianist. Fortunately there were plenty of patrons for his music and publishing houses anxious to commission his works. He was much sought after as a drawing-room pianist and popular teacher by the aristocratic families of Vienna. He went on concert tours to Berlin and Prague. Meanwhile at composition he worked very slowly and meticulously, usually three or four works at the same time. Sometimes he saw a work as a whole at the start, at other times a theme was written in his sketch book and stored away, but there were always many corrections before he was satisfied and ideas from early works often were used again much later.

Biographers have divided his adult life into three decades. In the first he had great social and professional success with many friends, always in love with some Court beauty, some of his favourites being Amalie Sebald, Giullietta Guicciardi, and the sisters Thérèse and Josephine von Brunsvik. His men friends included his teacher, Franz Ries, and all the best musicians of the day. He became engaged to Thérèse Malfatti but she broke this off in 1810. His early compositions showed originality of form, harmony, and key modulations but above all of dynamics; the sudden fortepianos, sforzandos and pianissimos being characteristic. His youthful sonatas for piano and small instrumental groups showed the influence of Mozart and Haydn but expressed more variety of mood and new technical features. His awareness of suffering, love and tenderness in his childhood home; virtue and goodness in his teachers and close friends; an overwhelming interest in people and his involvement in the new philosophy of the rights of man became an obsession to express these ideas powerfully through music. It has been said that his lack of facility for writing compared with his predecessors might have been due to his inconsistent early musical education. However, it can be postulated that this haphazard and unorthodox training gave him freedom to develop his own musical ideas, his rebellious nature being only moderately curbed by the kindly discipline of Neefe. The dramatic and turbulent quality of his works with rebellion against established forms and the sublime cantabile themes would only have come from richly varied experience. As a pianist his own technique gave tremendous effect to his style especially in some of the lovely slow movements.

### Musical Development

In 1800 the 1st Symphony in C (opus 21) astounded the musical world with its unusual slow opening movement, modifications of sonata form, and extensive use of brass instruments. From then onwards Beethoven became the acknowledged master of the symphony. The next eight years was a prolific period when his works included most of the quartets and the piano sonatas. Stimulated by his violinist friend Schuppanzigh, the sonatas for violin and piano were published in which it is noticeable that the pianoforte is of equal importance with the violin, thus reflecting his own skill as an accompanist. Other important works of the first decade included the ballet music to Prometheus in 1801, the first six symphonies, works for cello, the Mass in C Major, Opus 86, (1808), the Violin Concerto in D (Opus 61), a variety of chamber music and the piano concertos. His only opera, Fidelio, was performed in 1807 and was not at first a success.

Among the many characteristic features which began to emerge, two in particular are noteworthy, the lively scherzos and beautiful slow movements. A work which particularly illustrated the development of the symphony, the powerful and dramatic presentation and ability to manipulate in a new way old musical forms is the Eroica Symphony (No. 3 in E flat, Opus 55) first performed in 1805. This was dedicated originally to Napoleon whom Beethoven idolised because of his obsession with the equality and brotherhood of man and the suppression of tyrannical wealth and power. Napoleon shattered these ideals when he declared himself Emperor of France. The title page of "The Eroica" was torn up and the symphony rededicated to Prince Lobkowitz, one of Beethoven's most helpful patrons. The first movement with the striking horn chords and long development of the principal themes, the lively scherzo replacing the traditional elegant minuet, and the beautiful effects created by 3 horns in the trio all showed Beethoven's new ideas.

The Appassionata sonata for piano Opus 57 (F minor) was written in 1804. The reviewer wrote "In the first movement he has once again let loose many evil spirits . . . but as to the Adagio he said . . . I say that if you do not feel such music to go from heart to heart, one of us has none!" This wonderful slow movement showed the development of the theme and variations form. Many of the piano sonatas were dedicated to his friends, e.g. The Moonlight Sonata, Opus 27, to Giullietta Guicciardi, and the Waldstein, Opus 53.

Two great tragedies were milestones of his adult life which he passed with great pain. The clouds began to gather in his late twenties when he was often ill with fever, abdominal pain and diarrhoea. In his 26th year, he became aware of buzzing and singing in his ears, and soon after 30 the tragedy of his deafness was confirmed when he was walking with his friend and teacher Ries in the countryside and was unable to hear the high pure tones of the shepherd's recorder.

He reacted violently against his deafness, became depressed and went to the country where he considered suicide as shown in the famous Heiligenstadt Testament written to his brother in 1802. "Ah, how could I possibly admit an infirmity in the **one sense** which I once possessed in the highest perfection, a perfection such as few in my profession enjoy or ever have enjoyed . . . Forced to become a philosopher in my 28th year, Oh it is not easy, and for the artist much more difficult than for anyone else—Divine one, thou knowest that there dwells the love of mankind and the desire to do good". From his acquaintance with suffering in childhood there emerged this passionate urge to give his art to the world when he himself became afflicted.

Even in childhood Beethoven had been absorbed by the idea of "Fate", this always pursued him and became the motif of the 5th symphony, in C minor, Opus 68, so appropriately started at this turning point in his life. Fate for Beethoven always had to be wrestled with and evil invariably turned to good.

Beethoven was baptised a Roman Catholic but did not continue with any orthodox religion. However, his very deep religious feeling is shown in many letters and certainly he believed fervently in God, as the Creator and Father of all mankind. Compared with other great composers of his century his religious works were few and attempts to express his faith did not reach a climax until the great works of his last decade.

In the middle decade other symphonies emerged, the 5th and 6th were completed in 1808. The 6th (Pastoral) Symphony, Opus 68 is a reminder of Beethoven's intense love of nature and the impression made on him by natural phenonema, the storm, the brook, the bird songs. "I have fun like a child. What joy in wandering through the meadows among trees and flowers. It seems to me impossible that anyone can love nature as I do".

**Plate XXII. Bird songs from Pastoral Symphony: first movement.**

Increasing deafness, now believed to be due to otosclerosis, cut short his ambition as a pianist and orchestral conductor so that he turned his tremendous energy to composition. Goethe, who was one of Beethoven's heroes wrote in 1812 "I have never seen an artist more concentrated, energetic and fervent".

Beethoven's finances were always in a precarious state, dependent so much on the patronage of wealthy courtiers. For part of this middle period they became more stable with an assured income from his admirers, but poverty and financial disorder eventually overtook him again.

Perhaps the hardest problem for Beethoven was the isolation from human company created by his deafness. He was ashamed of his infirmity. In his youth he enjoyed the company of philosophers, students, poets; now he felt cut off and resorted to his conversation books, and wrote many letters to friends. The most famous of these is the love letter to "The immortal beloved", written in 1812. The unsolved mystery remains as to whom of his close woman friends this epithet applied. Amalie Sebald and Thérèse or Josephine von Brunsvik have often been mentioned but it seems more likely that this was the ideal woman of Beethoven's dreams with the best characteristics of his friends, and probably his mother as well, but that he never found this dream image and hence never married.

The last decade began with involvement again in family troubles. His brothers had both come to settle in Vienna, Casper Anton Carl as a civil servant and property owner, and Nicholas Johann in a pharmacy business; both were married and prosperous but Beethoven disapproved of their wives. Carl died in 1815 and made Beethoven the guardian of his only son, Karl. The boy's mother objected strongly but after a long drawn out legal battle, Beethoven gained his custody, and took the boy to live with him. He developed an intense love and possessiveness of this child, perhaps arising out of the image he still retained of his loving mother, and referred to him "As my precious son" signing himself "Your loving father". This perhaps showed the composer's desire for love and companionship of which in his almost complete deafness he felt so deprived, so once more he assumed the mantle of responsibility for family affairs, but Karl was an unsatisfactory ward, of mediocre intelligence, and a spendthrift. He wished to go into the army whilst his uncle planned an academic career for him. His debts reduced Beethoven to poverty and his behaviour caused him much sorrow.

As this last decade wore on Beethoven was almost completely deaf, introverted, only able to converse on paper, but working in a frenzied way for long hours as he was aware that he had still so much to create and his finances were precarious. The height of his intense emotion, and the depth and grandeur of his intellect continued to increase to the end, and in his last works he dispensed with all traditional form.

Since boyhood, Schiller's poetry had an immense appeal to him and his ambition was to set the "Ode to Joy" to music. This was Beethoven's creed for which he was unable to find adequate musical expression until his last years. After many years the ninth symphony was completed in 1824, and the last magnificent choral movement expresses Beethoven's religion of joy in the brotherhood of man. Schmidt says "His moral and religious intention was to reveal the power of joy to bring mankind together, and to show that it is rooted in the divine. Thus follows from the last of his symphonies all the ardour of his spirit which from his youth onward was moved by everything high and noble".

**Plate XXIII. Beethoven writing the Missa Solemnis.**

The Missa Solemnis (Opus 123) in D Major with its caption "From the heart to the heart of all men" also was completed in the same year and in its majesty and beauty as a religious work stands only next to J. S. Bach's B Minor Mass which was not then known to Beethoven. His achievement continued with the last piano sonatas and finally the last five great quartets and the Grosse Fuge, Opus 133, originally written as the last movement of the Quartet in B flat, Opus 130, but which, as it was too long was published as a separate work. Some critics suggest that these quartets should be regarded as one continuous whole.

From his early youth, Beethoven's work had that quality exemplified by Carlyle's dictum, "Genius means transcendent capacity for taking trouble". His last 10 years were an astonishing achievement for a man who suffered serious and painful illness much of the time and was almost totally deaf.

The last blow struck in July 1826 when his nephew Karl attempted suicide by shooting himself through the temporal regions whilst at the university. Fortunately the boy recovered, so was allowed to discontinue his studies and joined the army. He ultimately married and led a satisfactory life. Meanwhile Beethoven became more seriously ill with commencing liver failure. Whilst in the country convalescing at his brother Johann's house, he wrote to his old friend Dr. Wegeler recalling the kindness, friendship and good times of their youth in Bonn. "It is my hope yet to bring several great works into the world and then like an aged child, to end my earthly days among kindly companions" (Anderson).

In November 1826 his last two quartets to be published before his death, Opus 131 in C sharp minor, which Beethoven cosidered his greatest work, and Opus 135 were handed over to the publishers. The words of my title were written over the last movement of Opus 135. The first movement of Opus 131 expresses the depths of human suffering and later in these two works is the peace and joy that is experienced when this is over (example: Allegretto con amabile of Opus 131). In these last quartets is seen the greatness and complexity of Beethoven's life and his mastery over fate.

Beethoven's long illness with its varied clinical features of iridocyclitis, arthritis, gastro-intestinal symptoms, relapses and remissions is now thought to have been disseminated lupus erythematosus with lupus hepatitis leading to liver failure and terminal pneumonia (Larkin 1970). In March 1827, surrounded by physicians and friends he died dramatically in a thunderstorm with his arm raised to heaven.

As we enjoy these last great works of Beethoven, the dilemma can surely be answered, "Must it be? Yes, it must be", for "Genius does what it must"! (Meredith).

**Acknowledgements**

In the preparation of this lecture a great debt of gratitude is owed to several people: to Miss Liselotte Leshke for help with German translation; to Mr. Philip Moore, Head of Music, B.B.C. South and West, and Mr. David Gibbs of the Physics Department, University of Bristol for assistance with tape recording: Librarian, Stadt Archiv, Bonn for loan of slides; To Mr. Siegfried Brandenberg of Beethoven Archiv, Bonn for biographical advice. The illustrations are reproduced by courtesy of Beethovenhaus, Bonn.

**Musical Illustrations**

C. P. E. Bach. Solfeggietto.
J. S. Bach. Fugue in C major. The Well-Tempered Klavier. No. 1.
Ludwig van Beethoven:
- Theme and Variations on a March by Dressler in C minor. (Wo O 63).
- Piano Sonata No. 1 in E major (Wo O 47) 1st movement.
- Symphony No. 1 in C major (Op. 21) 1st movement.
- Sonata for Violin and Piano in F major (Op. 24) 1st movement.
- Symphony No. 3 in E major, Eroica (Op. 55) Scherzo: Allegro vivace.
- Symphony No. 6 in F major, Pastoral (Op. 68). Bird songs from 1st movement.
- Piano Sonata No. 23 in F minor, Apassionata (Op. 57) Andante con moto.
- Symphony No. 9 in D minor (Op. 125). "Ode to Joy".
- Piano Sonata in A flat (Op. 110). Ist movement played on Graf piano. Beethovenhaus, Bonn.
- Grosse Fuge in B flat. (Op. 133).
- String Quartet in C sharp minor (Op. 131) Allegretto con amabile.

**Bibliography**

Apley, John (1964) Lancet. **ii,** 1.
James, Burnett (1960) Beethoven and Human Destiny. Phoenix House, London.
Larkin, E. (1970) in Beethoven, the last Decade 1817-27. Cooper, M. Oxford University Press, London.
Meredith, Owen, from "Last Words of a Sensitive Second-rate Poet".
Tennyson, Hallam, (1970) History of Medicine, **2,** 20.
The following works have been extensively consulted and all other quotations are derived from them:—
Anderson, Emily (1961) The Letters of Beethoven. Macmillan, London.
Breuning, Gerhard von (1874) Aus dem Schwartzspanierhause, Vienna.
Cramer, C. F. (1783) Magazin der Musik, 1, 394.
McArdle, D. W. (1949); Musical Quarterly, **35,** 528.
Schiedermair, Ludwig (1925) Der junge Beethoven; Quelle Meyer. Bonn & Leipzig.
Schindler, Anton (1840); Biographie von Ludwig van Beethoven, 1st edition, Münster.
Schmidt, Leopold (1909) Beethoven Briefe, Berlin.
Schmidt-Görg, Joseph and Schmidt, Hans (1970); Ludwig van Beethoven. Deutsche Gramophon Gesselschaft, Hamburg.
Thayer, A. W., edited by Forbes, Elliott (1964) Life of Beethoven, Princeton University Press, New Jersey.
Wegeler, Franz and Ries, Ferdinand (1838); Biographische Notizen über Ludwig van Beethoven. Badeker, Coblenz.

## Figures and Tables

**Plate XX. f1:**
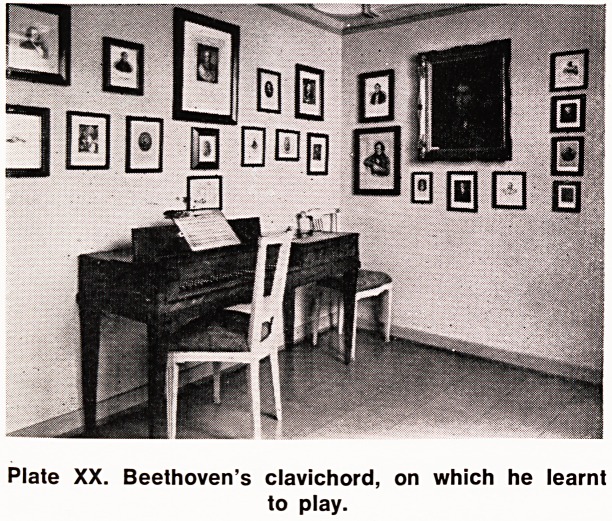


**Plate XXI. f2:**
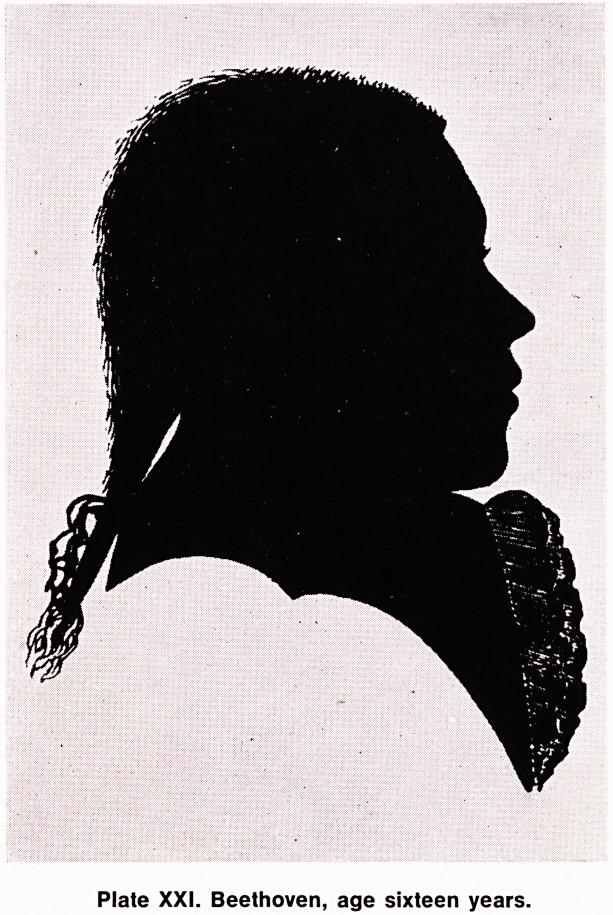


**Plate XXII. f3:**
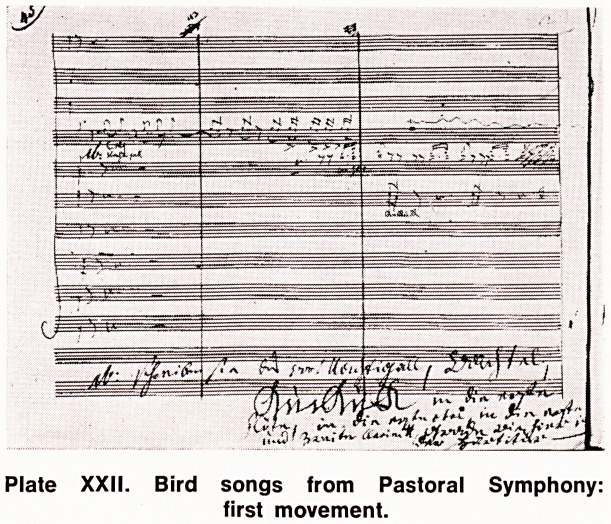


**Plate XXIII. f4:**